# Decandrinin, an unprecedented C_9_-spiro-fused 7,8-*seco*-*ent*-abietane from the Godavari mangrove *Ceriops decandra*

**DOI:** 10.3762/bjoc.10.23

**Published:** 2014-01-27

**Authors:** Hui Wang, Min-Yi Li, Félix Zongwe Katele, Tirumani Satyanandamurty, Jun Wu, Gerhard Bringmann

**Affiliations:** 1 Key Laboratory of Marine Bio-resources Sustainable Utilization, South China Sea Institute of Oceanology, Chinese Academy of Sciences, 164 West Xingang Road, Guangzhou 510301, P. R. China; 2 Hubei Key Laboratory of Natural Products Research and Development, College of Chemistry and Life Science, China Three Gorges University, Yichang 443002, P. R. China; 3 Marine Drugs Research Center, College of Pharmacy, Jinan University, 601 Huangpu Avenue West, Guangzhou 510632, P. R. China; 4 Institute of Organic Chemistry, University of Würzburg, Am Hubland, Würzburg 97074, Germany; 5 Government Degree College at Amadala valasa, Srikakulam District, Andhra Pradesh 532185, India

**Keywords:** abietane, absolute configuration, *Ceriops decandra*, circular dichroism, decandrinin, Rhizophoraceae

## Abstract

Decandrinin (**1**), an unprecedented C_9_-spiro-fused 7,8-*seco-ent*-abietane, was obtained from the bark of an Indian mangrove, *Ceriops decandra*, collected in the estuary of Godavari, Andhra Pradesh. The constitution and the relative configuration of **1** were determined by HRMS (ESI) and extensive NMR investigations, and the absolute configuration by circular dichroism (CD) and optical-rotatory dispersion (ORD) spectroscopy in combination with quantum-chemical calculations. Decandrinin is the first 7,8-*seco*-*ent*-abietane.

## Introduction

*Ceriops decandra* is a mangrove of the family Rhizophoraceae. It is widely distributed along the sea coasts of South Asia down to the southern pacific islands, and of Africa and Madagascar. The genus *Ceriops* only consists of five mangrove plant species. Besides *C*. *decandra*, these are *C. australis*, *C. pseudodecandra*, *C. tagal*, and *C. zippeliana* [1–4]. In Indian traditional medicine, the bark of *C. decandra* have been used for the treatment of amoebiasis, diarrhea, hemorrhage, and malignant ulcers [5], making it rewarding to screen the bioactive compounds of this plant. Before our work, already 28 compounds had been isolated from *C. decandra* [6] (three pimaranes, four beyeranes, five kauranes, and 16 lupanes). Recently, some of us have reported on the isolation of eleven new diterpenes from this plant, named decandrins A–K [7], of which nine belong to the group of abietanes.

**Figure 1:**
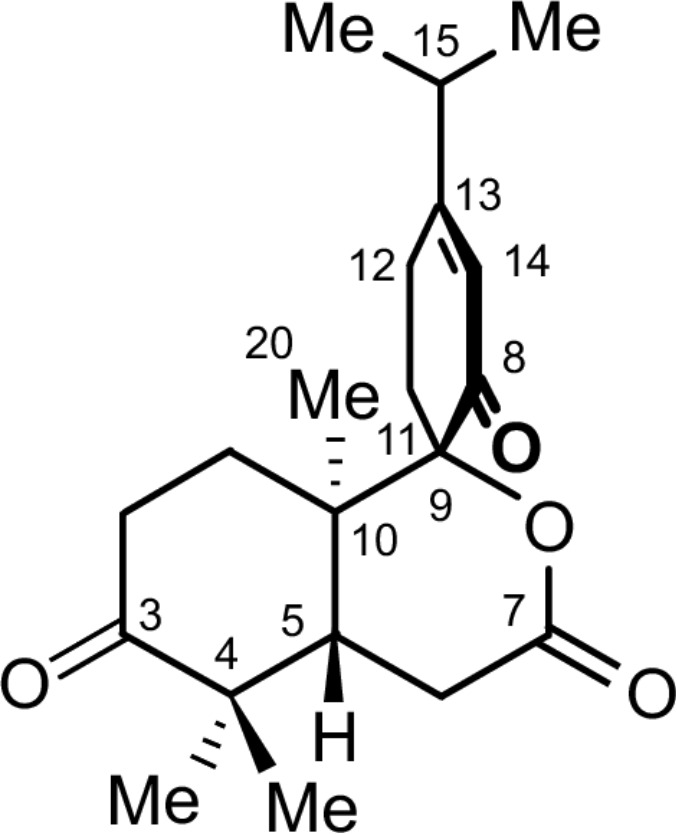
Structure of decandrinin (**1**).

S*eco-*abietane diterpenoids are a small group of natural products. To date, a total of 58 such compounds have been reported from plants of the genera *Abies*, *Cephalotaxus*, *Colus*, *Cordia*, *Hyptis*, *Isodon*, *Pinus*, *Premna*, *Salvia*, *Taiwania*, *Thuja*, and *Vitex*, including a 1,2-*seco*-abietane [8], a 1,10-*seco*-abietane [9–10], three 2,3-*seco*-abietanes [8,11–13], three 3,4-*seco-*abietanes [8,14], 31 4,5-*seco*-abietanes [8,15–16], ten 6,7-*seco*-abietanes [8,17–19], two 7,8-*seco*-abietanes [8,20], two 8,14-*seco*-abietanes [21–22], three 9,10-*seco*-abietanes [22–23], and two 9,11-*seco*-abietanes [24–25]*.* Among the above *seco*-abietanes, only laxiflorin V is a *seco*-*ent*-abietane [14]. Herein, we report on the isolation and structural elucidation of an unprecedented C_9_-spiro-fused 7,8-*seco*-*ent*-abietane, named decandrinin (**1**) (Figure 1), from the bark of an Indian mangrove, *C. decandra*, collected in the estuary of Godavari, Andhra Pradesh. The absolute stereostructure of **1** was established by HRMS (ESI), extensive NMR investigations, and by circular dichroism (CD) and optical-rotatory dispersion (ORD) spectroscopy in combination with quantum-chemical calculations.

## Results and Discussion

Decandrinin (**1**) was obtained as a colorless solid. Its molecular formula was established as C_20_H_28_O_4_ by HRMS (ESI) (*m*/*z* 333.2053, calcd for [M + H]^+^, 333.2060). From this formula, it was suggested that **1** has seven degrees of unsaturation, of which four could be ascribed to one carbon–carbon double bond, one lactone carbonyl group, and two ketone groups, according to its ^1^H and ^13^C NMR data (Table 1); the molecule should thus be tricyclic.

**Table 1:** ^1^H (400 MHz) and ^13^C (100 MHz) NMR spectroscopic data for **1** in CDCl_3_ (δ ppm).

Position	δ_H_ (*J* in Hz)	δ_C_

1α	1.87, m	31.5, CH_2_
1β	2.03, m	
2α	2.33, m	33.8, CH_2_
2β	2.68, m	
3		213.1, C
4		47.1, C
5	2.47, m	43.1, CH
6α	2.66, m	28.8, CH_2_
6β	2.47, m	
7		170.9, C
8		195.9, C
9		88.3, C
10		39.0, C
11α	2.59, m	30.1, CH_2_
11β	2.23, m	
12	2.52, m	26.6, CH_2_
	2.52, m	
13		172.0, C
14	6.03, br s	124.1, CH
15	2.45, m	35.4, CH
16	1.11, d (6.9)	20.6, CH_3_
17	1.11, d (6.9)	20.8, CH_3_
18	1.06, s	25.4, CH_3_
19	1.13, s	21.6, CH_3_
20	1.37, s	14.9, CH_3_

The NMR data and a DEPT experiment (Table 1) indicated the presence of an olefinic methine group [δ_H_ 6.03 (br s), δ_C_ 124.1], two aliphatic methine groups [δ_H_ 2.47 (m), δ_C_ 43.1; δ_H_ 2.45 (m), δ_C_ 35.4], five methylene groups [δ_H_ 2.33 (m), 2.68 (m), δ_C_ 33.8; δ_H_ 1.87 (m), 2.03 (m), δ_C_ 31.5; δ_H_ 2.59 (m), 2.23 (m), δ_C_ 30.1; δ_H_ 2.66 (m), 2.47 (m), δ_C_ 28.8; δ_H_ 2.52 (2H, m), δ_C_ 26.6], five methyl groups [δ_H_ 1.06 (3H, s), δ_C_ 25.4; δ_H_ 1.13 (3H, s), δ_C_ 21.6; δ_H_ 1.11 (d, *J* = 6.9 Hz, 3H), δ_C_ 20.8; δ_H_ 1.11 (d, *J* = 6.9 Hz, 3H), δ_C_ 20.6; δ_H_ 1.37 (3H, s), δ_C_ 14.9], two keto groups (δ_C_ 213.1, 195.9), and a lactone carbonyl group (δ_C_ 170.9). The NMR spectroscopic data indicated that **1** was a rearranged abietane.

**Figure 2:**
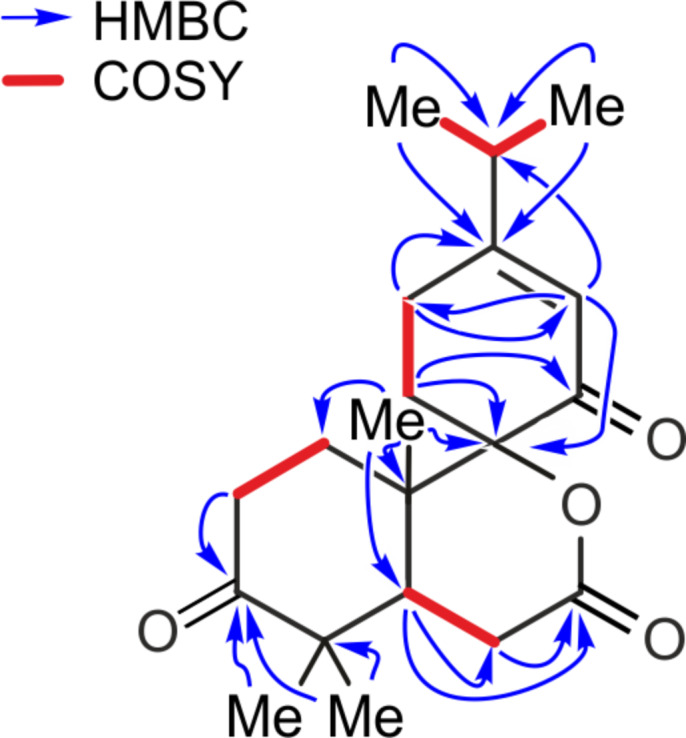
Selected ^1^H–^1^H COSY and HMBC correlations for decandrinin (**1**).

The existence of an isopropyl group was suggested by ^1^H,^1^H-COSY correlations between H-15 and protons of two methyl groups [δ_H_ 1.11 (d, *J* = 6.9 Hz, 3H), 1.11 (d, *J* = 6.9 Hz, 3H)]. From ^1^H,^1^H-COSY correlations, three further proton–proton spin systems, viz. H_2_-1–H_2_-2, H_2_-11–H_2_-12, and H-5–H_2_-6, were deduced (Figure 2).

HMBC correlations between H_3_-16/C-13, H_3_-17/C-13, and H-14/C-15 placed the above isopropyl group at C-13, while those from H-14 to C-9 and C-12 indicated the presence of a ∆^13,14^ double bond. HMBC correlations from H_3_-18, H_3_-19, and H_2_-2 to the carbon at δ_C_ 213.1 suggested the location of a keto group at C-3, whereas those from H_2_-11 to the carbon at δ_C_ 195.9 indicated that there was another keto group at C-8 (Figure 2).

HMBC correlations from H-5 and H_2_-6 to the carbonyl carbon (δ_C_ 170.9) of a δ-lactone suggested its location at C-7, while those from H_2_-11, H-14, and H_3_-20 to the quaternary carbon (δ_C_ 88.3) placed it at C-9 (Figure 2).

**Figure 3:**
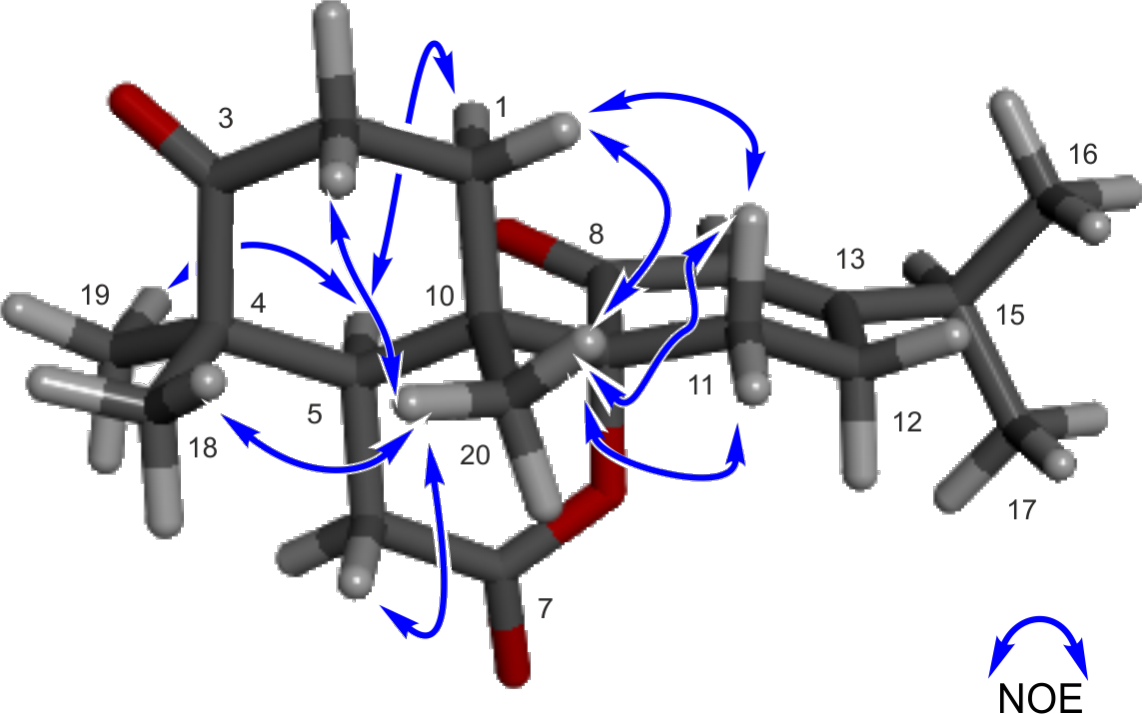
Diagnostic NOE interactions for decandrinin (**1**, B97D/TZVP-optimized structure): arbitrarily the 5*R*,9*R*,10*S*-enantiomer is shown.

The NOE interactions for the two methyl groups at C-4 suggested that one methyl group is located at the same side as H-5, while the other one has the same orientation as Me-20. The NOEs between the two protons of the methylene at C-11 and Me-20 led to the conclusion that the carbonyl at C-8 is opposite to Me-20 (Figure 3). If the carbonyl at C-8 was oriented in the same direction as Me-20 these NOEs would not be observed because there would be several atoms between the concerned protons (Figure S9 in Supporting Information File 1). Therefore, the relative configuration of **1** was identified as shown in Figure 1.

**Figure 4:**
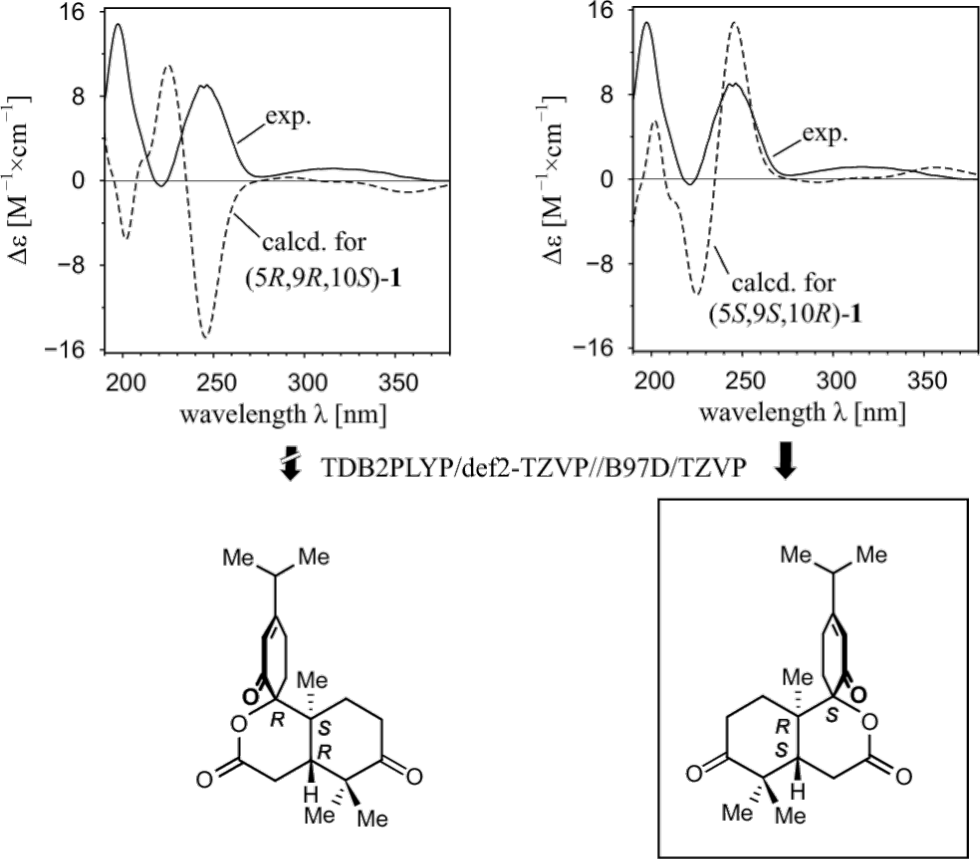
Determination of the absolute configuration of decandrinin (**1**) by comparing the calculated CD spectra with the experimental one.

The absolute configuration of **1** was assigned by CD and ORD spectroscopy in combination with quantum-chemical calculations. The conformational analysis of **1** by using RI-SCS-MP2/def2-TZVP//B97D/TZVP yielded six relevant conformers within the energetical range of 3 kcal/mol above the global minimum. For each of the six conformers thus identified, TDB2PLYP/def2-TZVP calculations were performed providing single UV and CD spectra, which were then summed up with Boltzmann weighting. The resulting averaged CD spectrum was corrected by a UV shift [26] of 13 nm and compared with the experimental CD curve (Figure 4). While the CD curve predicted for the 5*R*,9*R*,10*S*-configuration was nearly opposite to the one experimentally observed, the spectrum calculated for the 5*S*,9*S*,10*R*-enantiomer showed a good fitting with a moderate Δ_ESI_ value of 58% [27]. To further corroborate the assignment of the absolute configuration of **1**, ORD calculations were performed using the PBE0/cc-pVDZ//B97D/TZVP method. The ORD calculated for the 5*S*,9*S*,10*R*-configuration in the non-resonant region matched with the one observed experimentally (Figure S10 in Supporting Information File 1). The good agreement of the experimental CD and ORD spectra with the ones calculated for the 5*S*,9*S*,10*R-*enantiomer revealed the absolute configuration of **1** to be as shown in Figure 4.

**Scheme 1:**
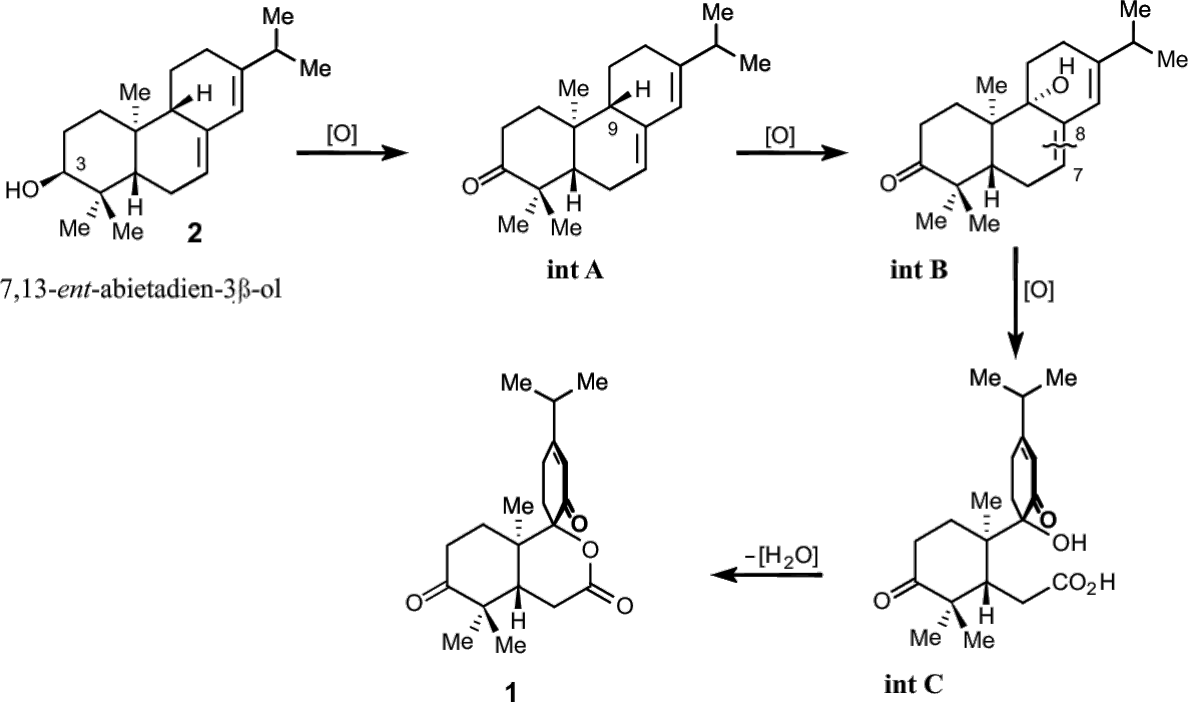
Proposed biosynthetic pathway for decandrinin (**1**).

A plausible biogenetic precursor of decandrinin (**1**) might be the naturally more common β-diastereomer of 7,13-*ent*-abietadien-3-ol (**2**). Accordingly, its 3β-OH group would be oxidized to yield **int A**, whose C-9 would then be hydroxylated to afford **int B**. Oxidative cleavage at the ∆^7,8^ double bond of **int B** could yield **int C**. Finally, the lactonization of **int C** would give decandrinin (**1**) (Scheme 1).

## Experimental

### General methods

Optical rotation values were recorded on a JASCO P-1020 polarimeter. CD spectra were recorded on a J-715 spectropolarimeter (JASCO, Gross-Umstadt, Germany). UV spectra were obtained on a Beckman DU-640 UV spectrophotometer. NMR spectra were recorded on a Bruker Avance 400 NMR spectrometer in CDCl_3_. High-resolution ESI mass spectra were performed on a Bruker maXis UHR-TOF mass spectrometer in positive ion mode. For column chromatography, silica gel (200–300 mesh, Qingdao Mar. Chem. Ind. Co. Ltd.) and RP C_18_ gel (YMC) were used. High-performance liquid chromatography (HPLC) was performed on a Shimadzu LC-6AD controller with an SPD-20A UV–vis detector equipped with YMC-Pack ODS-A columns (250 × 10 mm i.d., 5 μm and 250 × 4.6 mm i.d., 5 μm).

### Plant material

As described previously [7] the bark of *Ceriops decandra* were collected in September 2009 in the estuary of Godavari, Andhra Pradesh, India. The identification of the plant was performed by one of the authors (T.S.). A voucher sample (No. CD-001) is maintained at the Marine Drugs Research Center, College of Pharmacy, Jinan University.

### Extraction and isolation

The extraction and isolation procedures were in part identical to those described recently [7]: The chloroform extract (65.2 g) from air-dried bark (7.4 kg) of *C. decandra* was subjected to silica-gel column chromatography (200−300 mesh, 3.0 kg) and eluted with petroleum ether/acetone (100:0 to 1:2) to yield 285 fractions. Fractions 173 to 204 were combined and further purified using RP C_18_ column chromatography eluted with acetone/H_2_O (30:70 to 100:0) to give 57 subfractions. Subfractions 8–13 were combined and subjected to preparative HPLC (YMC-Pack 250 × 10 mm i.d., MeCN/H_2_O, 32:68) to afford eight subfractions. Then the sixth subfraction was further purified by HPLC (YMC-Pack 250 × 4.6 mm i.d., MeOH/H_2_O, 40:60) to provide **1** (1.9 mg).

### Characterization

Decandrinin (**1**): Colorless solid; [α]_D_^25^ +242.0 (*c* 0.12, Me_2_CO); UV (MeCN) λ_max_ 246.9 nm; For ^1^H and ^13^C NMR spectroscopic data (see Table 1); HRMS (ESI) *m*/*z*: [M + H]^+^ calcd for C_20_H_29_O_4_, 333.2060; found 333.2053.

### Computational details

The B97D/TZVP [28–29] method was used to perform the conformational analysis of **1** with Gaussian 09 [30]. Single-point energy calculations at the RI-SCS-MP2/def2-TZVP [31–32] level and TDB2PLYP/def2-TZVP [33–35] calculations in combination with the COSMO solvation model with acetonitrile as a solvent and the chain-of-spheres approximations [35–37] were done using ORCA [38]. The optical rotatory dispersion (ORD) was calculated at the PBE0/cc-pVDZ [39–40] level using Gaussian 09. The Boltzmann weighting of single UV and CD spectra, UV shift, the determination of Δ_ESI_ values in the wavelength region between 190 nm and 380 nm (13 nm UV shift, σ = 0.22 eV), and the comparison of the calculated CD (σ = 0.22 eV) and ORD spectra with those observed experimentally were done with SpecDis 1.61 [41].

## Supporting Information

File 1 HRMS (ESI) and NMR spectra of decandrinin (**1**), NOE interactions for the B97D/TZVP-optimized structure diagnostic for the 9-epimer of decandrinin (**1**), and comparison of the calculated ORD with the experimental one.
