# Supplementary material for: Decandrinin, an unprecedented C9-spiro-fused 7,8-seco-ent-abietane from the Godavari mangrove Ceriops decandra
Source: Beilstein J Org Chem. 2014 Jan 27;10:276–81. doi: 10.3762/bjoc.10.23 (PMC3943825; doi:10.3762/bjoc.10.23)
Supplement: File 1 — HRMS (ESI) and NMR spectra of decandrinin (1), NOE interactions for the B97D/TZVP-optimized structure diagnostic for the 9-epimer of decandrinin (1), and comparison of the calculated ORD with the experimental one. [file Beilstein_J_Org_Chem-10-276-s001.pdf]

Supporting Information

for

Decandrinin, an unprecedented C_9-spiro-fused 7,8-*seco*-*ent*-abietane from the Godavari mangrove *Ceriops decandra*

Hui Wang[1,2], Min-Yi Li[3], Félix Zongwe Katele[4], Tirumani Satyanandamurty[5], Jun Wu*[,3] and Gerhard Bringmann*[,4]

Address: [1]Key Laboratory of Marine Bio-resources Sustainable Utilization, South China Sea Institute of Oceanology, Chinese Academy of Sciences, 164 West Xingang Road, Guangzhou 510301, P. R. China, [2]Hubei Key Laboratory of Natural Products Research and Development, College of Chemistry and Life Science, China Three Gorges University, Yichang 443002, P. R. China, [3]Marine Drugs Research Center, College of Pharmacy, Jinan University, 601 Huangpu Avenue West, Guangzhou 510632, P. R. China, [4]Institute of Organic Chemistry, University of Würzburg, Am Hubland, Würzburg 97074, Germany and [5]Government Degree College at Amadala valasa, Srikakulam District, Andhra Pradesh 532185, India

Email: Jun Wu* - wwujun2003@yahoo.com; Gerhard Bringmann* - bringmann@chemie.uni-wuerzburg.de

* Corresponding author

HRMS (ESI) and NMR spectra of decandrinin (1), NOE interactions for the B97D/TZVP-optimized structure diagnostic for the 9-epimer of decandrinin (1), and comparison of the calculated ORD with the experimental one.

Mass Spectrum SmartFormula Report

Analysis Info			
Analysis Name	D:\Data\MS\data\201211\wanghui_66_pos.d	Acquisition Date	11/28/2012 3:21:18 PM
Method	POS_100-2000_Dirrect Infusion.m	Operator	SCSIO
Sample Name	SCSIO	Instrument / Ser#	maXis 29
Comment			

Acquisition Parameter

Source Type	ESI	Ion Polarity	Positive	Set Nebulizer	0.3 Bar
Focus	Not active	Set Capillary	3500 V	Set Dry Heater	180 °C
Scan Begin	100 m/z	Set End Plate Offset	-500 V	Set Dry Gas	4.0 l/min
Scan End	2000 m/z	Set Collision Cell RF	2000.0 Vpp	Set Divert Valve	Waste

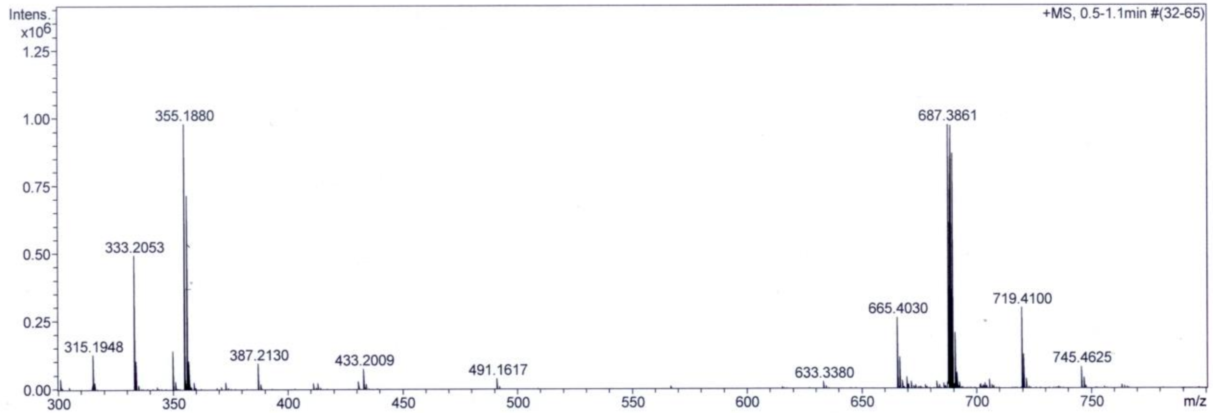

Meas. m/z	#	Formula	Score	m/z	err [mDa]	err [ppm]	mSigma	rdb	e¯ Conf	N-Rule
333.2053	1	C 20 H 29 O 4	100.00	333.2060	0.8	2.3	3.0	6.5	even	ok
355.1880	1	C 21 H 24 N 4 Na	100.00	355.1893	1.3	3.6	285.8	11.5	even	ok
	2	C 19 H 25 N 4 Na 2	23.78	355.1869	-1.1	-3.2	298.6	8.5	even	ok
	3	C 20 H 28 Na O 4	33.30	355.1880	-0.1	-0.2	300.2	6.5	even	ok
687.3861	1	C 40 H 56 Na O 8	100.00	687.3867	0.6	0.9	488.2	12.5	even	ok

Bruker Compass DataAnalysis 4.0 printed: 11/28/2012 3:29:33 PM Page 1 of 1

Figure S1: HRMS (ESI) spectrum of decandrinin (**1**) (MeOH)

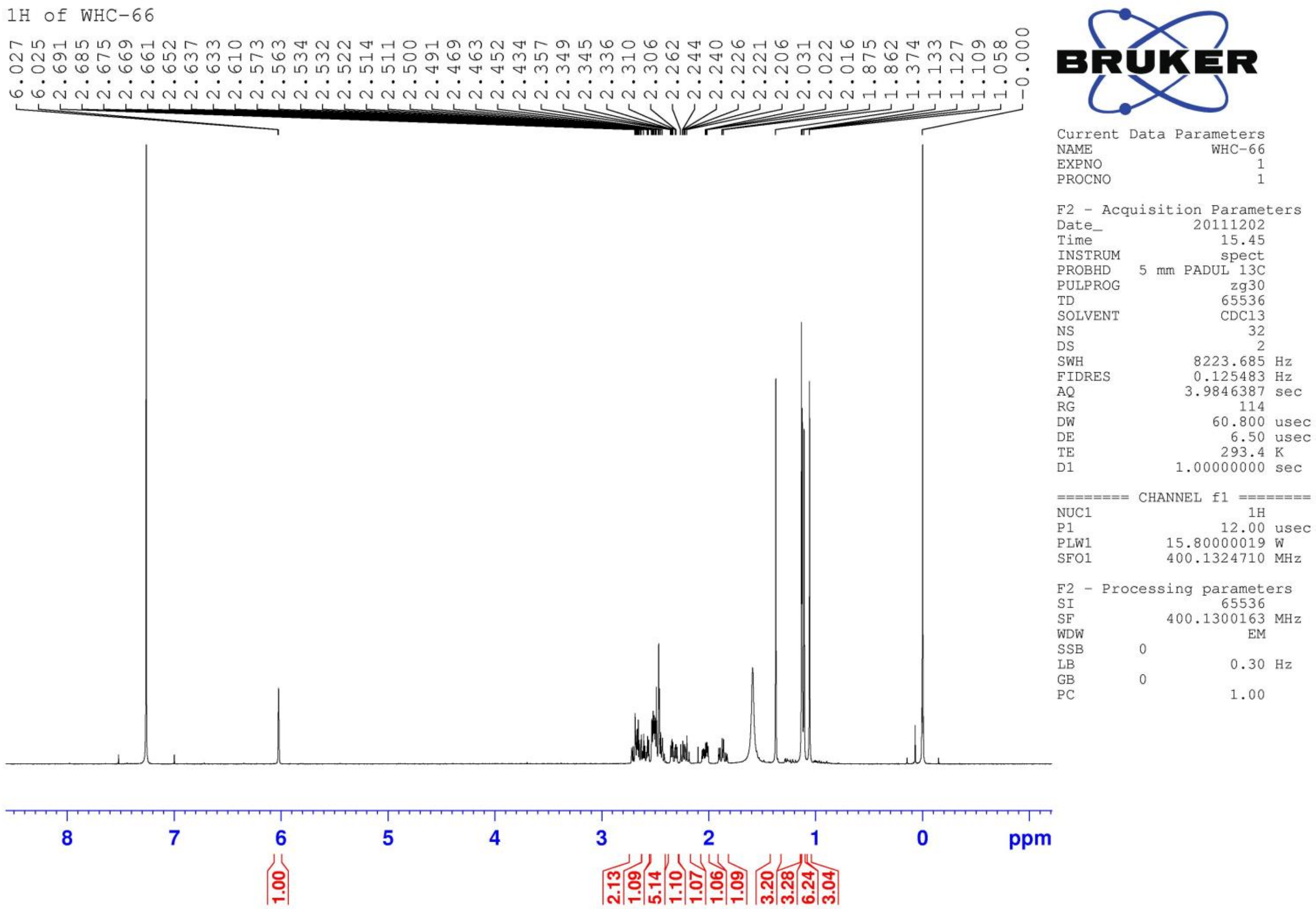

Figure S2A: ^{1}H NMR spectrum of decandrinin (**1**) (400 MHz, $CDCl_3$)

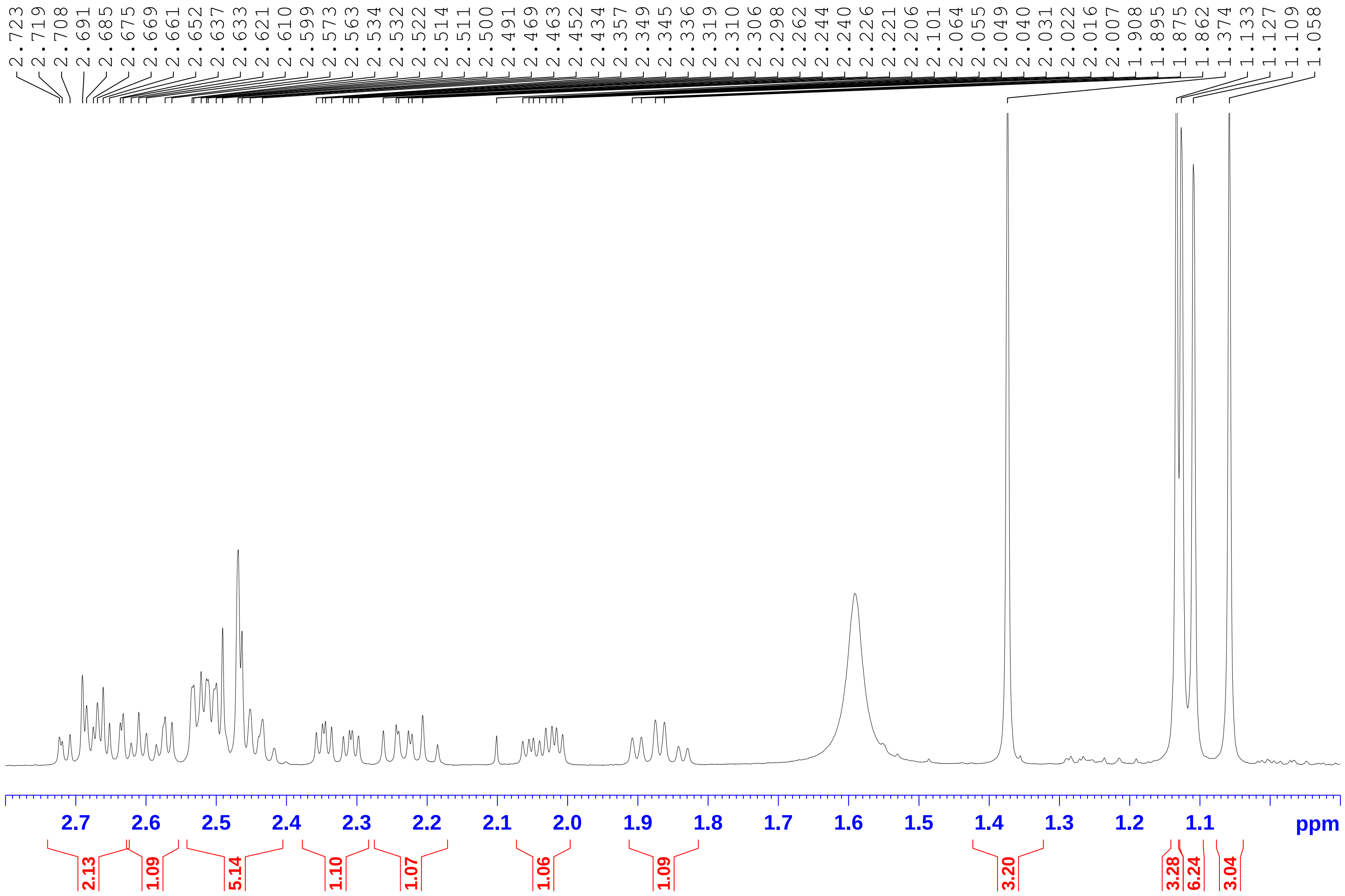

Figure S2B: Enlargement of area 1.0–2.7 ppm

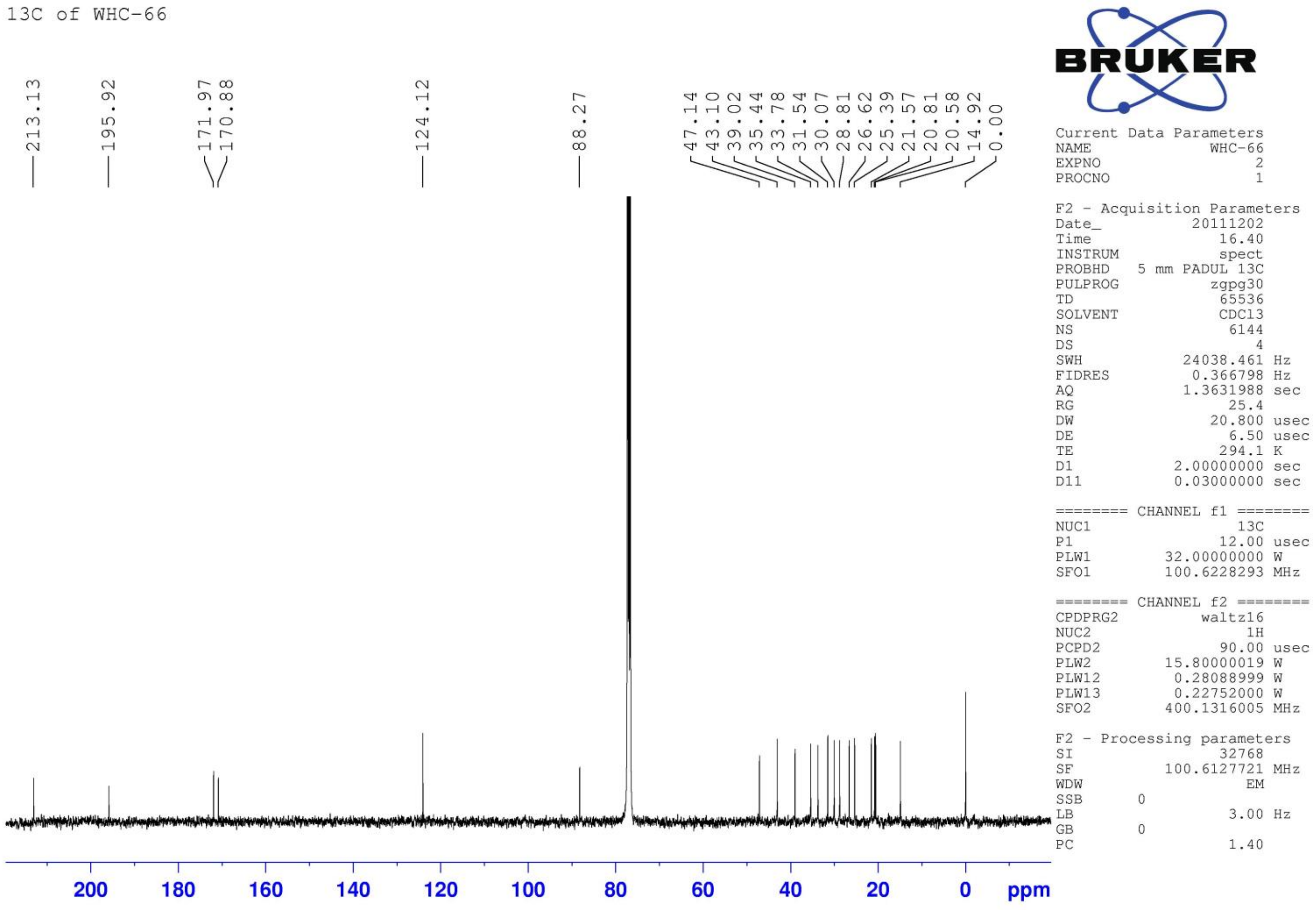

Figure S3: ^{13}C NMR spectrum of decandrinin (**1**) (400 MHz, $CDCl_3$)

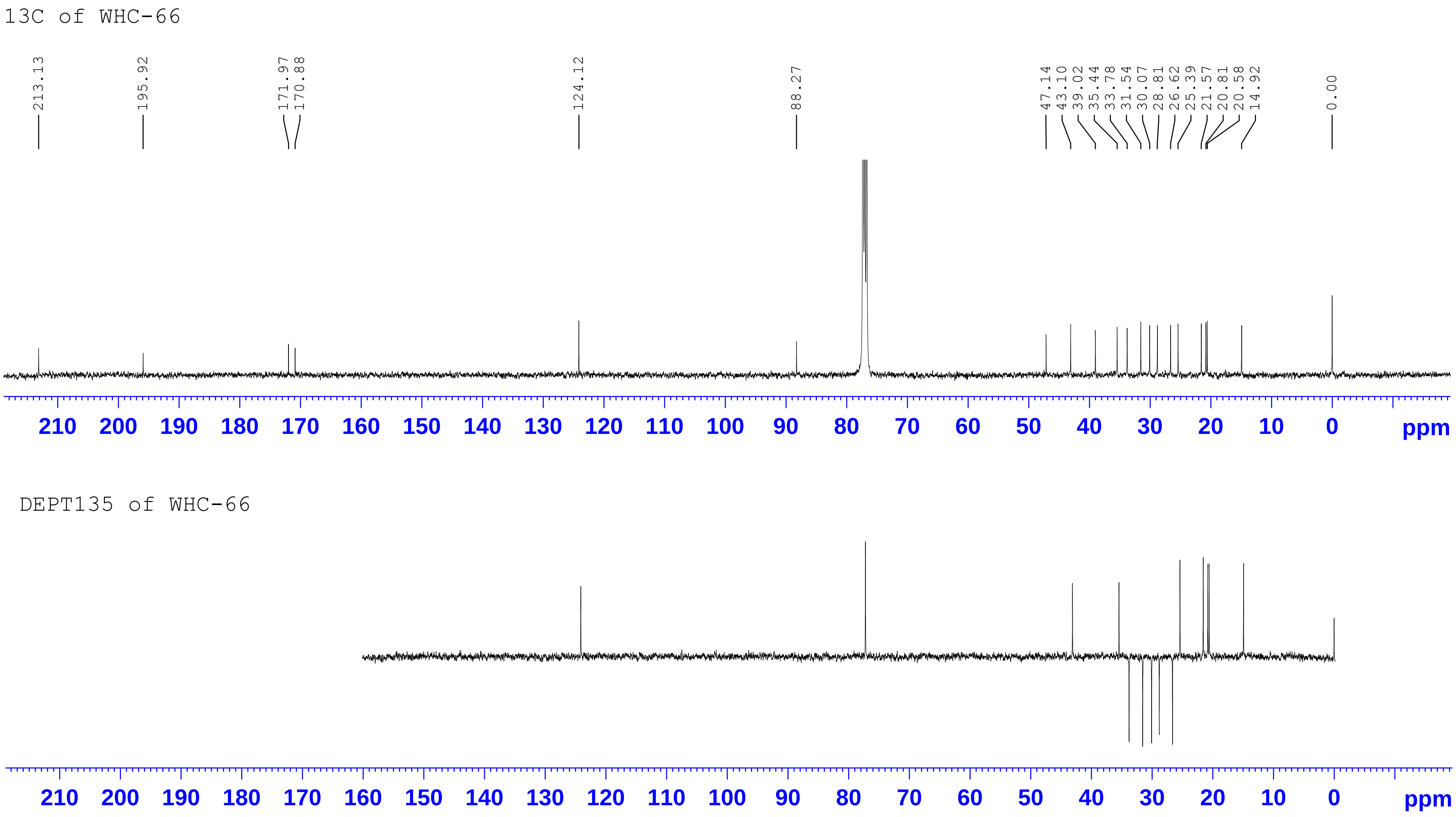

Figure S4: ^{13}C NMR and DEPT 135 spectra of decandrinin (**1**) (400 MHz, $CDCl_3$)

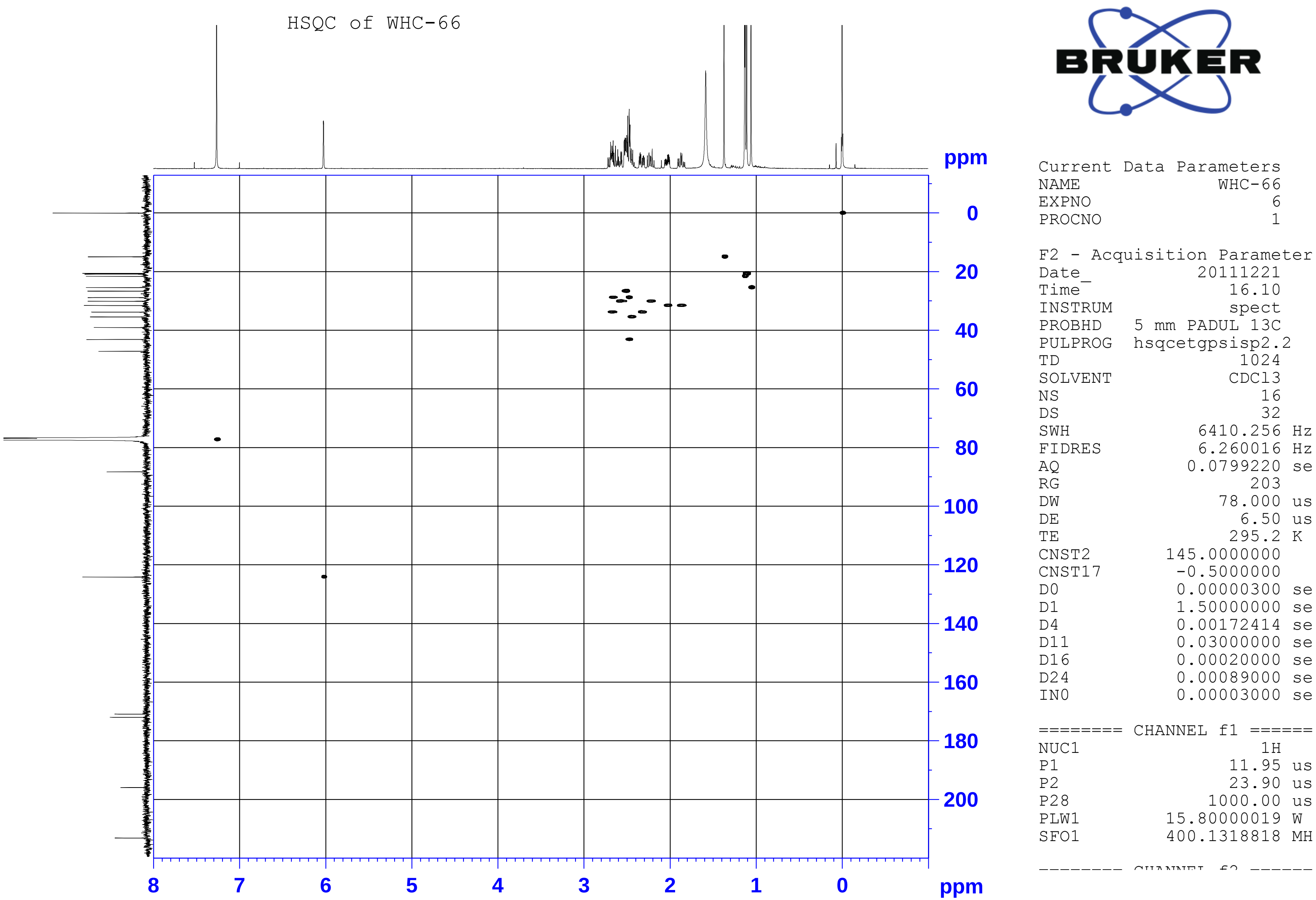

Figure S5A: HSQC spectrum of decandrinin (**1**) (400 MHz, $CDCl_3$)

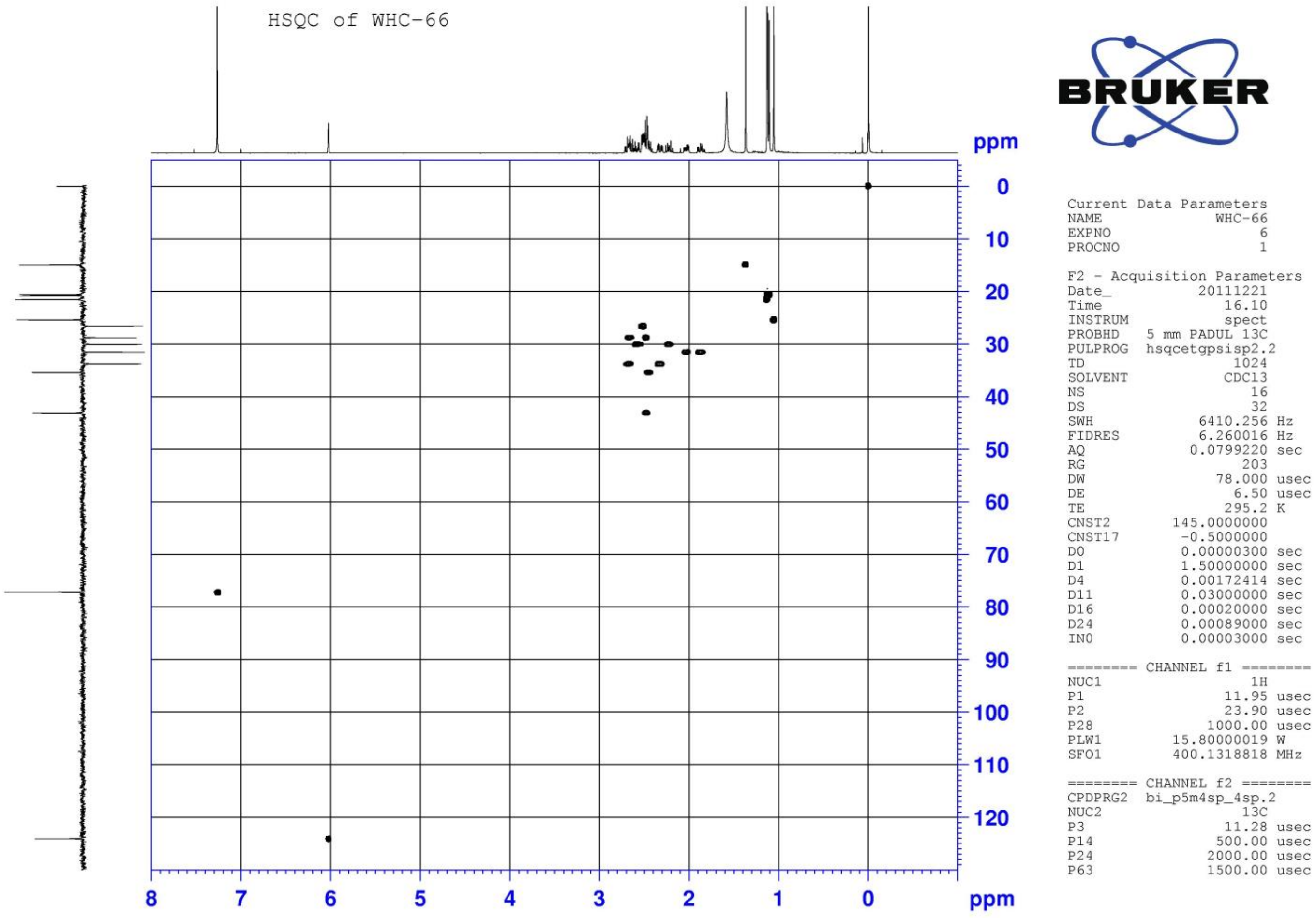

Figure S5B: Enlargement of area (^{1}H) 0.0–8.0 ppm and (^{13}C) 0–130 ppm

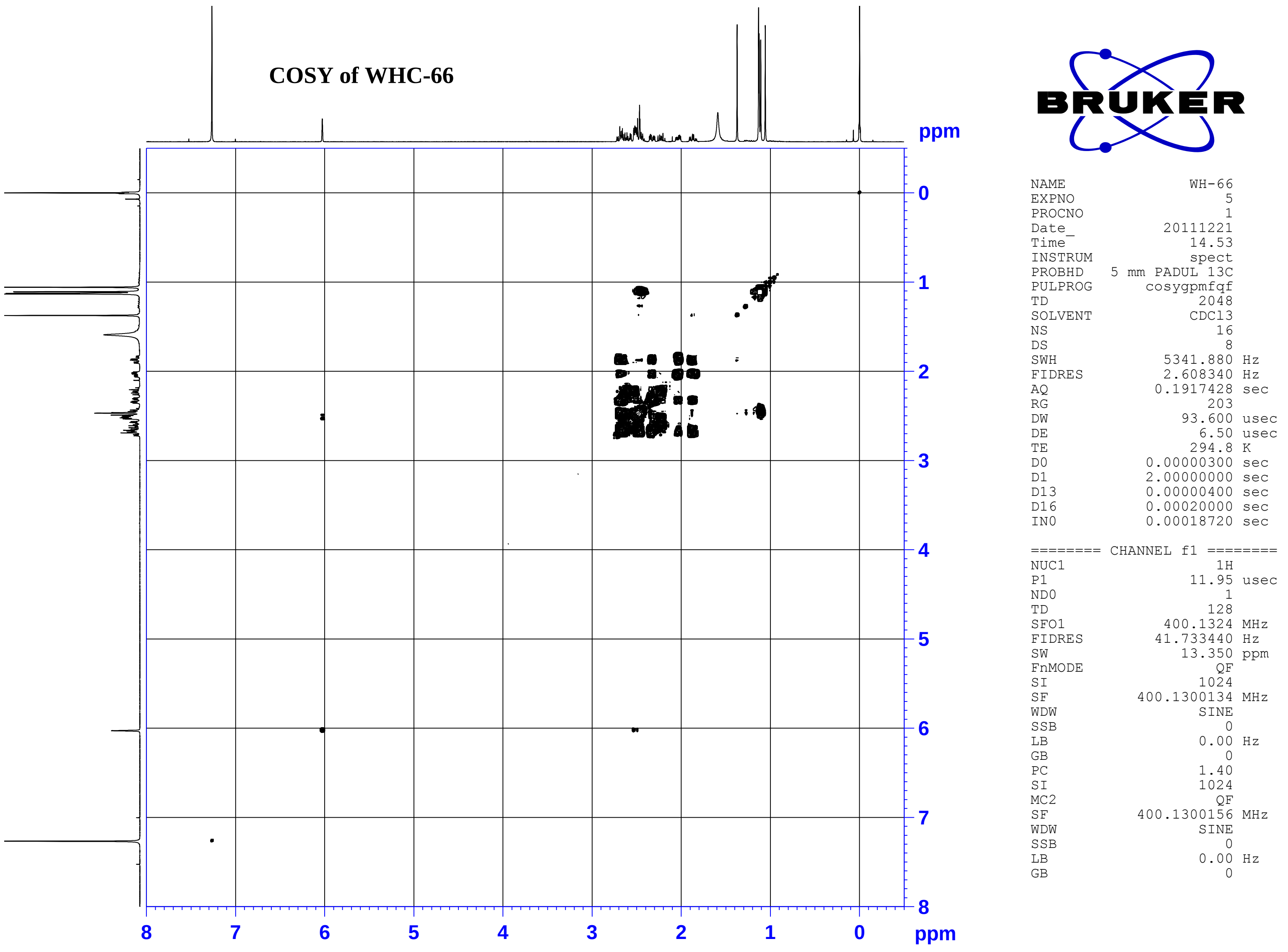

Figure S6A: $^{1}H,^{1}H$ COSY spectrum of decandrinin (**1**) (400 MHz, $CDCl_3$)

COSY of WHC-66

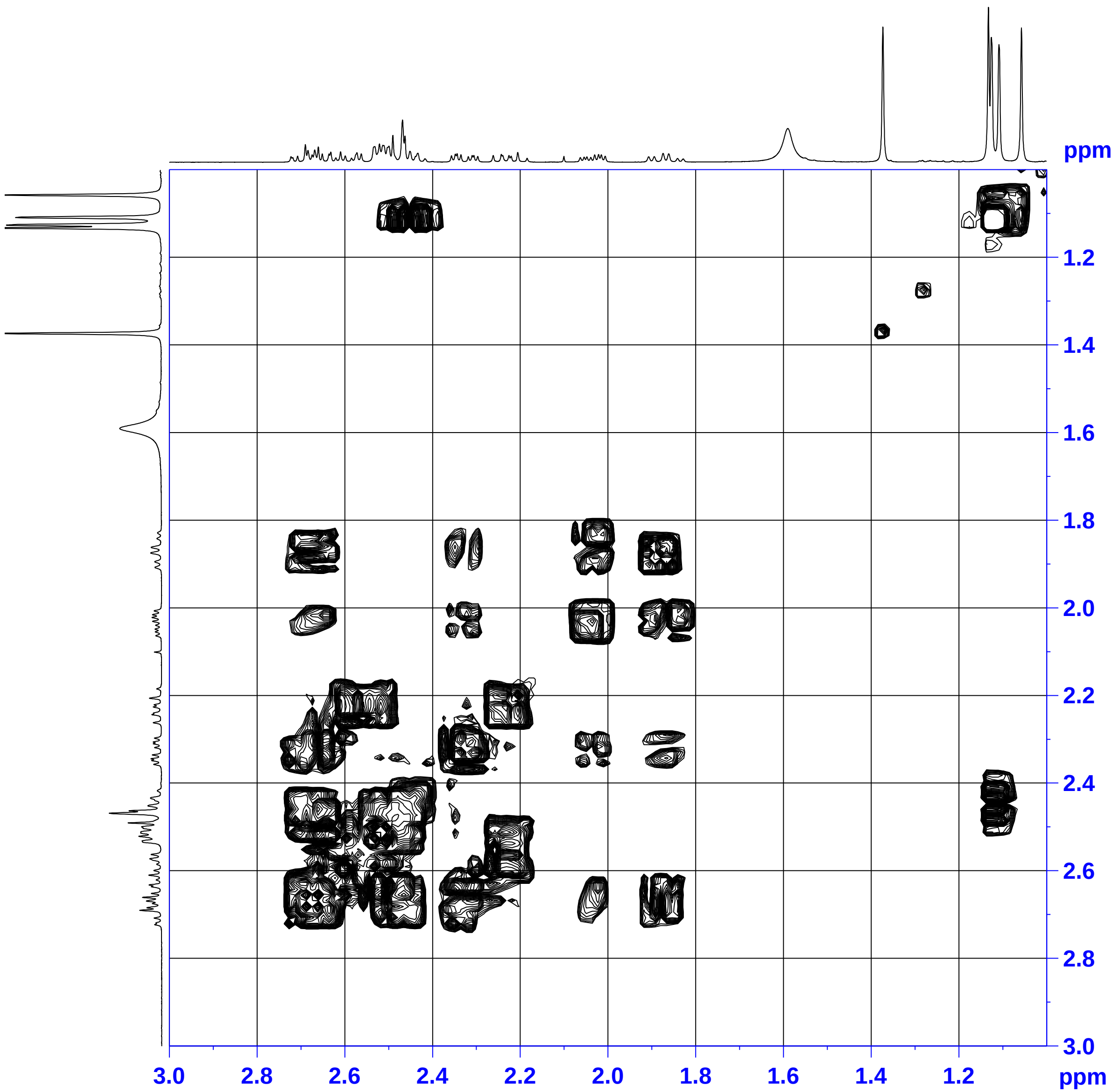

Figure S6B: Enlargement of area 1.0–3.0 ppm

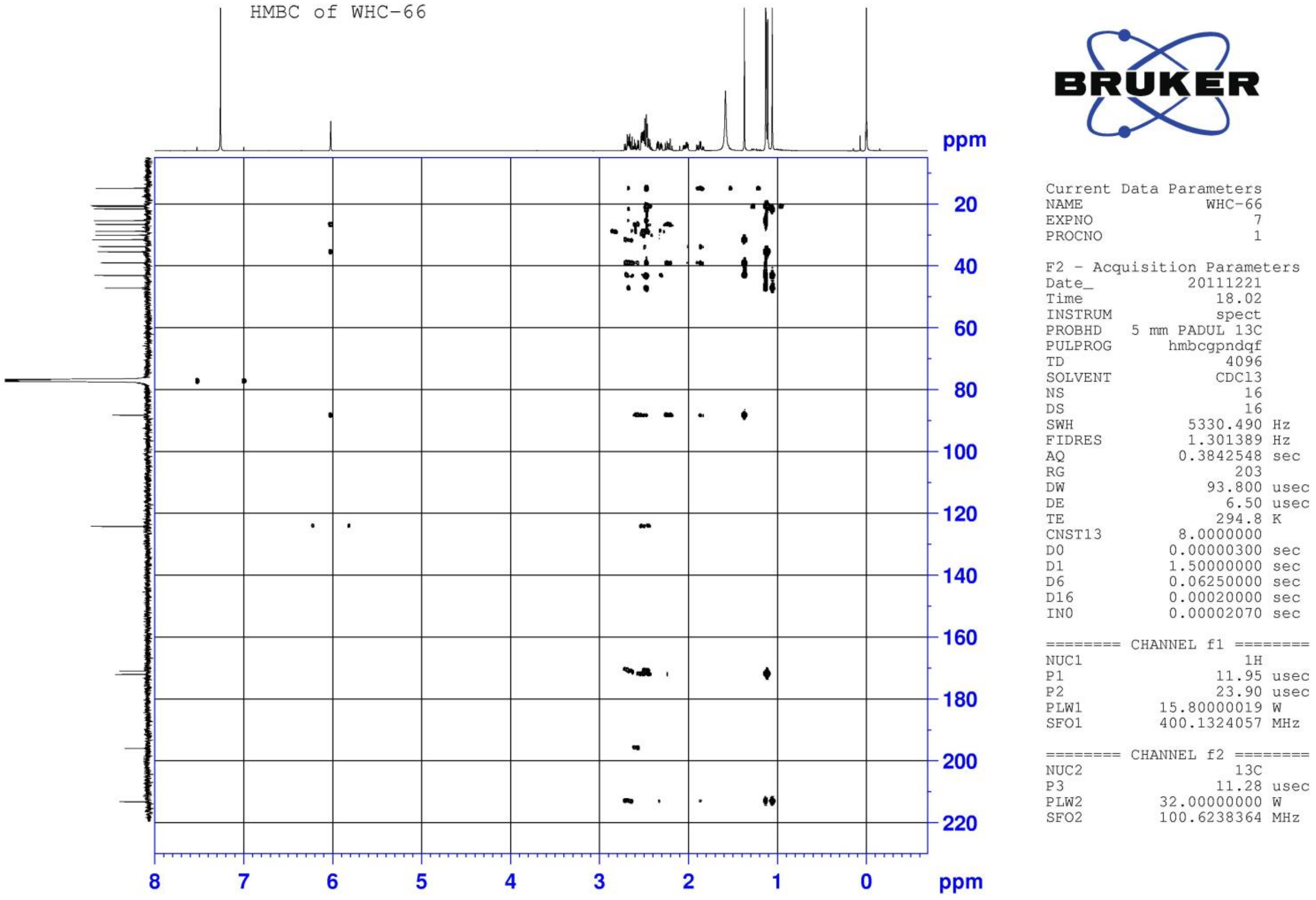

Figure S7A: HMBC spectrum of decandrinin (**1**) (400 MHz, $CDCl_3$)

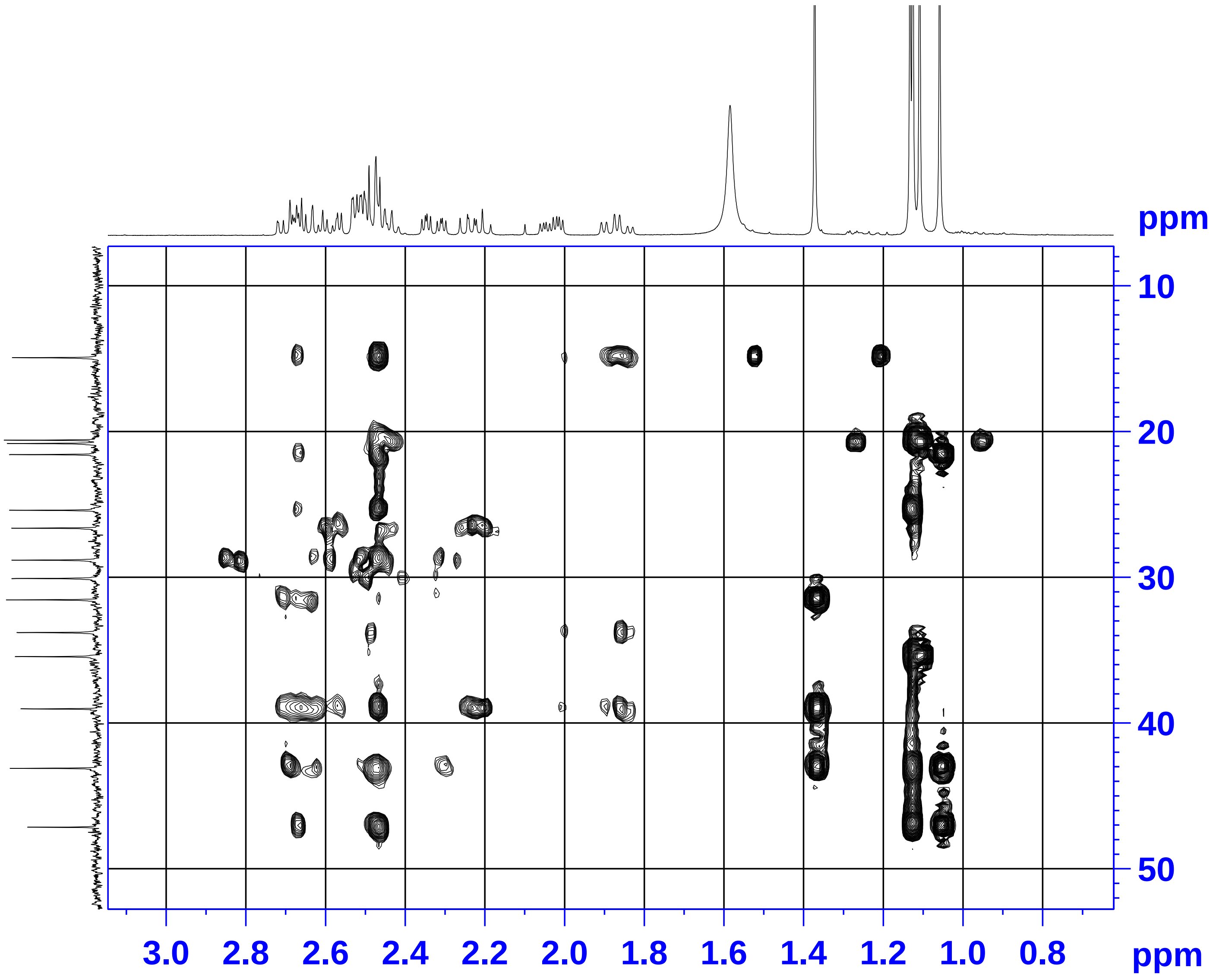

Figure S7B: Enlargement of area (^{1}H) 0.8–3.0 ppm and (^{13}C) 10–50 ppm

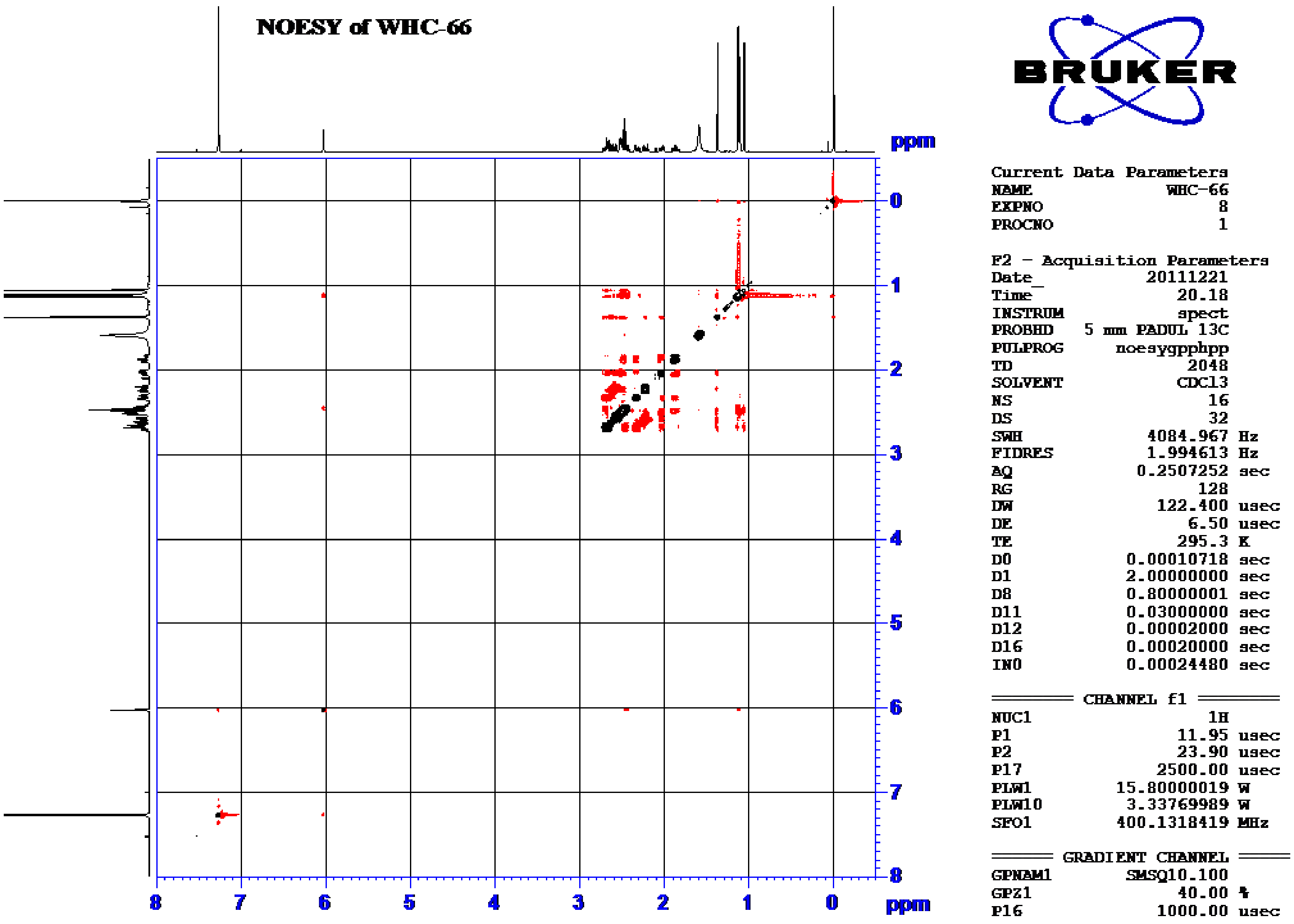

Figure S8A: NOESY spectrum of decandrinin (**1**) (400 MHz, $CDCl_3$)

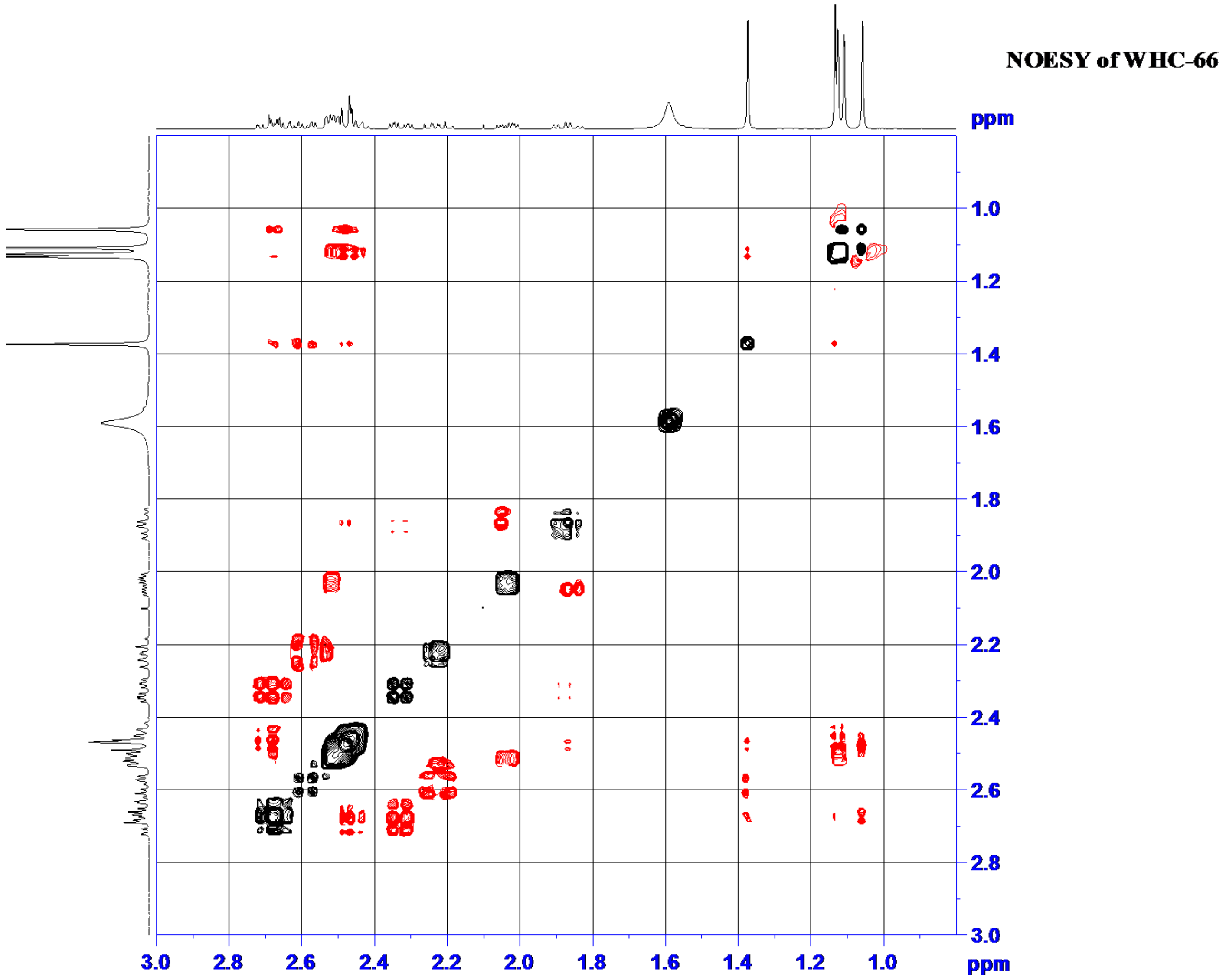

Figure S8B: Enlargement of area 1.0–3.0 ppm

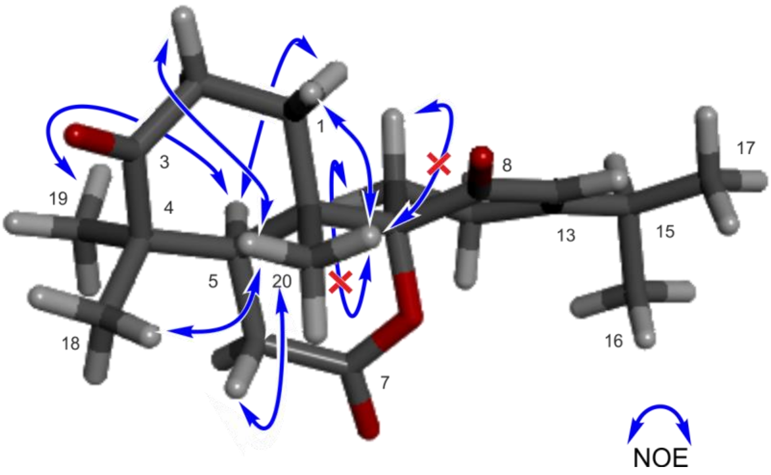

Figure S9: NOE interactions for the B97D/TZVP-optimized structure diagnostic for the (unnatural) 9-epimer of decandrinin (**1**): arbitrarily the 5*R*,9*S*,10*S*-enantiomer is shown.

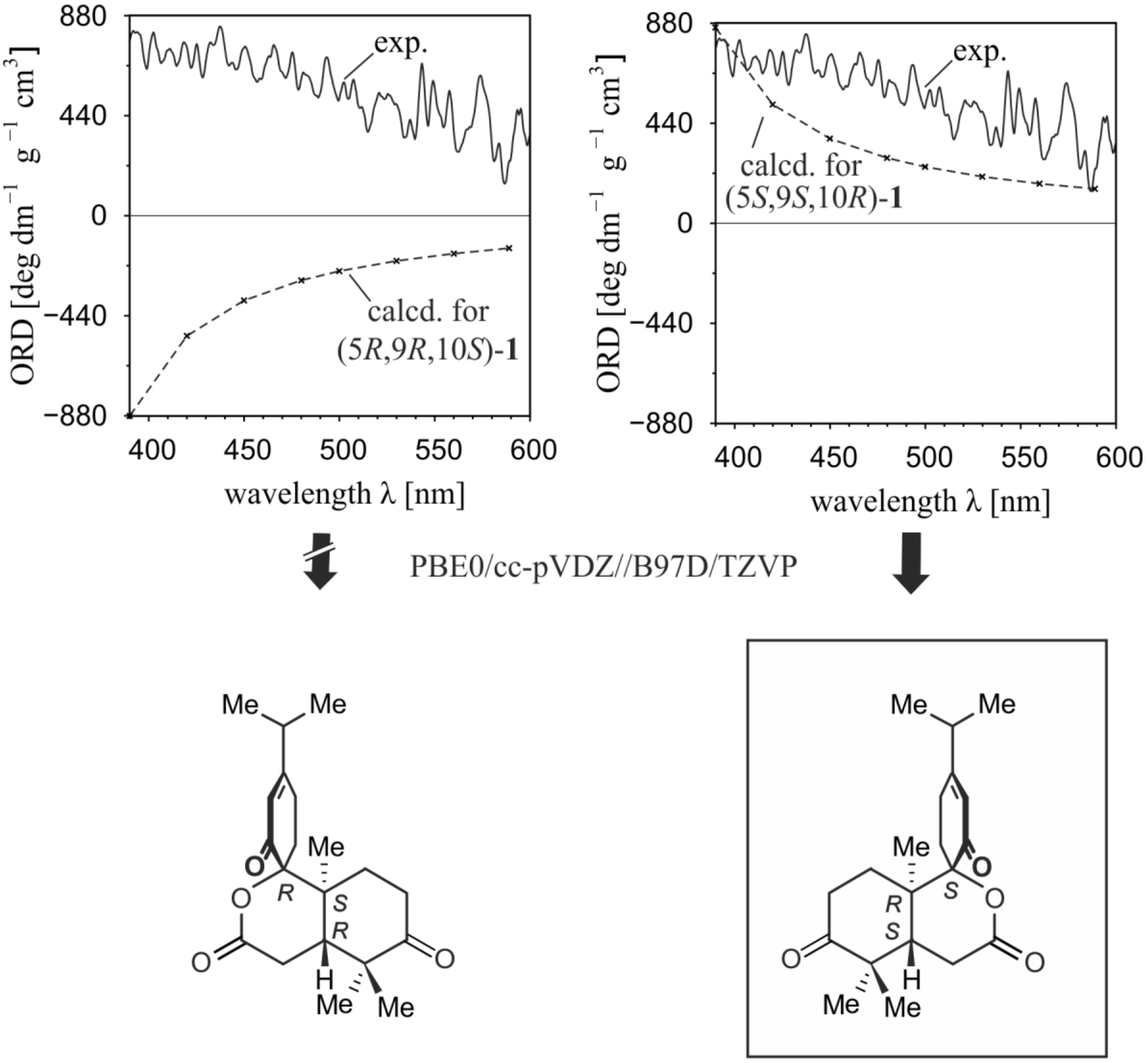

Figure S10: Confirmation of the absolute configuration of decandrinin (**1**) by comparison of the calculated ORD with the experimental one.
